# Technological Advancements in Augmented, Mixed, and Virtual Reality Technologies for Surgery: A Systematic Review

**DOI:** 10.7759/cureus.76428

**Published:** 2024-12-26

**Authors:** Ashley Y Sang, Xinyao Wang, Lamont Paxton

**Affiliations:** 1 Biomedical Engineering, Miramonte High School, Orinda, USA; 2 Biomedical Engineering, The Harker School, San Jose, USA; 3 Private Practice, General Vascular Surgery Medical Group, Inc., San Leandro, USA

**Keywords:** augmented reality (ar), classical machine learning, deep learning (dl), extended reality (xr), mixed reality (mr), surgery general, virtual reality (vr)

## Abstract

Recent advancements in artificial intelligence (AI) have shown significant potential in the medical field, although many applications are still in the research phase. This paper provides a comprehensive review of advancements in augmented reality (AR), mixed reality (MR), and virtual reality (VR) for surgical applications from 2019 to 2024 to accelerate the transition of AI from the research to the clinical phase. This paper also provides an overview of proposed databases for further use in extended reality (XR), which includes AR, MR, and VR, as well as a summary of typical research applications involving XR in surgical practices. Additionally, this paper concludes by discussing challenges and proposed solutions for the application of XR in the medical field. Although the areas of focus and specific implementations vary among AR, MR, and VR, current trends in XR focus mainly on reducing workload and minimizing surgical errors through navigation, training, and machine learning-based visualization. Through analyzing these trends, AR and MR have greater advantages for intraoperative surgical functions, whereas VR is limited to preoperative training and surgical preparation. VR faces additional limitations, and its use has been reduced in research since the first applications of XR, which likely suggests the same will happen with further development. Nonetheless, with increased access to technology and the ability to overcome the black box problem, XR’s applications in medical fields and surgery will increase to guarantee further accuracy and precision while reducing risk and workload.

## Introduction and background

In 2020, the artificial intelligence (AI) healthcare market was projected to reach US$ 6.6 billion by 2021, with a compound annual growth rate of 40% [1]. With rapid advancements in technology, various applications have begun to be utilized in medical fields to increase medical capabilities [2,3]. Starting as early as the 1980s, the pursuit of accuracy and precision led to the early development of robotic surgical systems. This was mainly seen through the preliminary development of robotic surgery, which showed the potential to minimize the risk of complications, reduce blood loss, and alleviate pain beyond human capabilities [4,5]. During the mid-2010s, research exploring the use of augmented reality (AR), mixed reality (MR), and virtual reality (VR) in medical surgery introduced new possibilities for the industry, with initial successes demonstrated in some cases [6,7]. In 2015, a study by Diana et al. on minimally invasive surgery using a combination of AR and robotic surgical systems highlighted that it could further increase safety by providing image guidance. This study also discussed how other AI technologies may be used to address those challenges [6]. Today, the purpose of robotic surgery remains the same but has advanced to allow AI pairings and has been shown to improve surgeon visualization, increase precision, and decrease fatigue [7]. Similarly, for preparation and training, three-dimensional printing (3DP) has significantly improved patient outcomes by allowing surgeons to create physical models that anticipate potential issues that may occur during the surgery [8,9]. When 3DP became commonly used in training and preoperative surgical planning, people turned to more advanced and detailed anatomical representations using VR, AR, and MR [10-12].

The growth of AI technology has resulted in widespread application across various industries, particularly in healthcare. This is largely because AI can solve many limitations of traditional surgery such as long operating times and higher error risks, offering great potential to enhance the precision and efficiency of procedures [13,14]. AI technology enables cognitive robots to understand their environments and learn from experience, improving their performance over time while developing customized treatment plans for each patient in fields such as oncology and orthopedics. This in turn leads to a faster treatment process and improves overall safety [13-16]. AI can also predict perioperative and postoperative complications by analyzing a patient’s medical history, genetic data, and biological and imaging parameters, minimizing the need for invasive screening methods [14,17,18]. This capability is exemplified in cardiovascular surgery, where machine learning (ML) models analyze echocardiograms to detect anomalies and evaluate heart function [19-21]. Similarly, in oncology, AI supports surgical planning, optimizes treatment approaches, and aids in the resection of lesions during surgery [22-26]. In neurosurgery, surgeons can navigate through intricate brain structures with high accuracy using image-guided surgery [27-30]. With AI assistance, surgeons can safely perform highly complex surgeries and reduce the risk of complications, which has led to the widespread adoption of AI in various medical functionalities.

This paper provides a comprehensive review of new advancements in AR, MR, and VR for surgical applications from 2019 to 2024, structured into four main sections. This paper first covers the growth of classical ML and deep learning in conjunction with extended reality (XR). Following this, the paper details developments of AR, MR, and VR within surgical practice. This paper also includes a summary of proposed databases valuable for continued XR research and highlights common applications of XR in surgical settings, concluding with an analysis of challenges and proposed solutions. The objective of this paper is to identify the growth of AR, MR, and VR alongside AI technology in the medical field.

## Review

Methodology

The articles discussed in this review were collected from databases such as PubMed, IEEE (Institute of Electrical and Electronics Engineers), ScienceDirect, and Springer using keywords and phrases, including “augmented reality”, “mixed reality”, “virtual reality”, “medical surgery”, “machine learning”, and “classical learning”. From there, only articles published within the past five years (2019 to present) were included to accurately reflect current developments in AR, MR, and VR applications in surgery. However, articles detailing the initial stages of development in the history of AR, MR, and VR surgical applications outside this five-year timeframe were also included to provide context for current developments and demonstrate a trend of advancement. Lastly, the articles in this review were thoroughly evaluated to ensure their relevance to AR, MR, and VR applications in surgery. A total of 9383 articles came as a result of our initial search, and 670 duplicate articles were removed. We screened 8498 articles and removed 8249 articles as they either were unrelated to the topic of this article or did not provide us with enough access to fully comprehend the article. In total, 249 articles were selected for retrieval, 245 of which were assessed for eligibility. Ultimately, 82 articles were selected from the initial pool identified during the search.

The datasets included in this review were sourced from Google Search, Google Scholar, and PubMed. These datasets consist of CT or MRI images of the human brain, chest, heart, lungs, spine, and knee. Seven of the datasets contain images of tumors in various body parts. The search for these databases was conducted using the keywords “CT” and “MRI”. There were no restrictions on the recency of the datasets, and both public and private datasets were considered. All datasets in this review are valuable for AR, MR, and VR surgical development, particularly for 3D segmentation purposes. Our initial search identified 2405 datasets. We screened 2302 datasets, 1989 of which were removed either due to a lack of credibility or data decay and 95 were duplicates. In total, 309 datasets were assessed for eligibility. In total, 18 datasets were selected for inclusion in the article from the initial search results.

**Figure 1:**
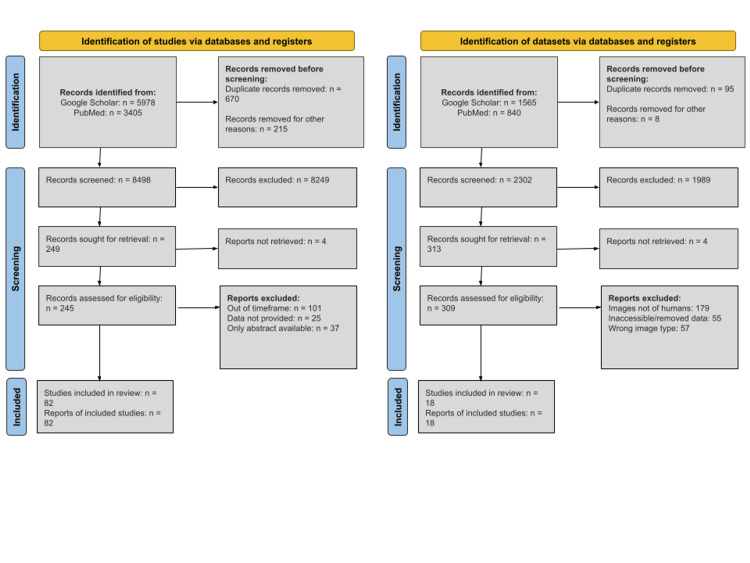
PRISMA flow diagrams for both included studies (left) and included datasets (right). PRISMA: Preferred Reporting Items for Systematic Reviews and Meta-Analyses.

All articles and database collections were conducted following the Preferred Reporting Items for Systematic Reviews and Meta-Analyses (PRISMA) guidelines. Figure 1 illustrates the search methods and inclusion criteria for articles and databases.

Review of datasets

Tables 1, 2 summarize proposed MRI and CT databases for XR research in biomedical fields, specifically for AI and ML developments. These tables include dataset names, sample sizes, body parts images, availability (public or private), and references. As high-quality, large sample size datasets are integral to the advancement of AI/ML technology, especially within medical fields, these datasets are intended to aid in the development of these technologies by ensuring credible, accurate, and helpful data.

**Table 1:** Summary of recommended MRI databases for AI/ML medical development. AI: artificial intelligence; ML: machine learning; MR: magnetic resonance; T1W: T1-weighted.

Dataset name	Sample size	Body part(s)	Availability	Ref.
Annotated MRI Images of Acute Stroke Patients	2888 MR images with clinical diagnosis of acute or early subacute stroke	Brain	Private	[31]
Deep Image Reconstructions of the Brain	86 TI-weighted images, precise segmentations of 12 brains	Brain	Public	[32]
Spinal Cord fMRI	T2-weighted images from 10 healthy participants, three neck positions: flexion, neutral, extension	Spine	Public	[33]
Cross-sectional MRI Data in Adults	416 subjects, 119 non-demented males, 197 non-demented females, 41 demented males, 59 demented females	Brain/Alzheimer's	Private	[34]
MRIs of Demented and Non-Demented Brains (OASIS)	150 subjects, 72 non-demented, 64 demented, 14 initially non-demented then diagnosed as demented, 3-4 T1-weighted MR images per subject	Brain/Alzheimer's	Private	[35]
fastMRI Dataset of Brains and Knees	8770 DICOM image files, 1,500 knees, 6,970 brains	Brain/Breast/Knee/Prostate	Private	[36-38]
Lumbar Spine MRI Dataset	515 subjects, 48,345 MRI slices - 320 x 320 pixels	Spine	Public	[39,40]
The Cancer Genome Atlas Low Grade Glioma Collection	199 subjects, 241,183 MR images	Brain/Cancer	Public	[41,42]
The Anatomical Tracings of Lesions after Stroke	955 T1-weighted MR images, 655 T1W MRIs and lesion masks, 300 T1W in a hidden dataset	Brain/Stroke	Private	[43-45]

**Table 2:** Summary of recommended CT databases for AI/ML medical development. AI: artificial intelligence; ML: machine learning; SqCCs: squamous cell carcinomas; MR: magnetic resonance.

Dataset name	Sample size	Body part(s)	Availability	Ref.
RAD-ChestCT Dataset	36,000 chest CT scans with a matrix of 84 abnormality labels x 52 location labels from 20,000 patients	Chest	Private	[46,47]
NLM The Visible Human Project	Male: 1,871 cross-sections for both CT and anatomical images. Female: 5,189 anatomical image	Head, neck, longitudinal sections of the rest of the body	Public	[48]
DeepLesion	32,735 lesions in 32,120 bookmarked CT slices from 10,594 studies of 4427 unique patients	Lung nodules, liver lesions, enlarged lymph nodes, kidney lesions, bone lesions, etc.	Public	[49]
TCGA-LUSC	178 lung SqCCs in addition to whole-genome sequencing (WGS) of 19 samples and mRNA sequencing of 159 samples	Lung cancer	Public	[50]
OASIS-3	55 cognitively normal adults and 622 individuals at various stages of cognitive decline ranging in age from 42 to 95 years, 2842 MR sessions, over 2157 raw imaging scans	Brain/Alzheimer's	Private	[51]
LUNA16	888 CT scans, divided into 10 subsets	Lung cancer	Private	[52]
Lung-PET-CT-Dx	CT resolution was 512 × 512 pixels at 1 mm × 1 mm, 355 subjects	Lung cancer	Public	[53]
COPDgene 1-10	10 case sets, 7298 anatomic landmarks paired between the 10 sets of images, pairs per case ranged from 447 to 1172	Heart and lungs	Private	[54]
CT Scan Images - Mendeley	238 cancerous images, 126 non-cancerous. Total 364 images.	Heart and lungs	Public	[55]

Classical learning vs. deep learning

**Figure 2:**
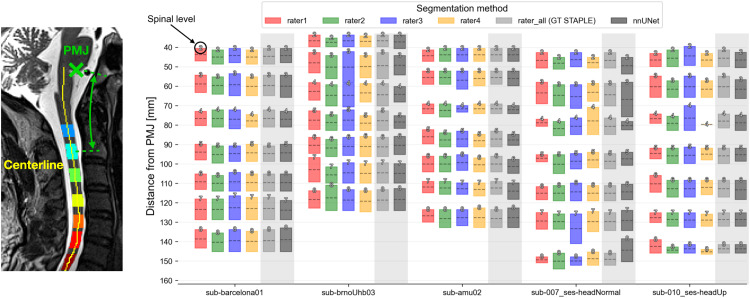
Segmentation, which is the division of an image to allow for the identification of certain objects, is an example of an artificial intelligence (AI) application in the medical field today. PMJ: pontomedullary junction. Source: Valosek et al. [33]. Used under the terms of the Creative Commons Attribution CC BY License.

Traditional classical learning has been rapidly surpassed by deep learning in recent years, driven by advancements in the developments of convolutional neural networks (CNN)/artificial neural networks (ANN) and visual simultaneous localization and mapping (VSLAM) technologies, which offer higher accuracy and are better suited for solving complex problems. The initial aim of AI applications in surgery was to assist novice surgeons with organ visualization and intraoperative guidance [56]. AI allows surgeons to project an image of the patient’s anatomy onto the body, facilitating more precise incisions [2,17,57]. Beginning in the 1990s, AI was also utilized for surgical training, as AI-generated simulations proved to be safe and effective training methods for novice surgeons [58]. In 2015, the Lancet Commission on Global Surgery reported that 18 million deaths were due to inadequate medical care [59]. This finding led to the release of a joint commission by The Lancet and the Financial Times focusing on the integration of AI, health information technology, and universal health access [60]. Soon after, the use of e-learning, ML, and VR in surgical training increased and was found to be more effective than traditional training methods [61-64]. As technology advanced throughout the 21st century, deep learning emerged as the preferred approach due to its superior problem-solving capabilities and accuracy, gradually replacing classical learning [65]. In more recent developments, where AI in surgery is more commonly used in image classification and identification, tool navigation, and virtual training, deep learning has proven to be more capable of addressing high-complexity problems that classical learning cannot (Figure 2) [66].

Augmented reality in surgery

Over the past five years, AR developments have focused on optimizing performance to make the technology faster, more accurate, and capable of generating graphics using deep learning models. Gupta et al. investigated whether deep learning could enhance media retrieval time in AR, achieving an average media retrieval time of 1.1471 μs and a client response time of 1.1207 μs [67]. Similarly, Lan developed an AR application to aid in surgical training, demonstrating that it was 10 times faster than methods using regional CNN, achieving a mean Jaccard score of 97.5 and a mean average precision (mAP) of 96.8 for tool detection [68]. Specifically, AR advancements in surgery over the last five years have focused on preoperative and intraoperative applications and have focused on improving surgical safety through increasing accuracy and precision, location detection, and navigation.

In terms of preoperative planning for surgery, AR development has largely focused on optimizing surgical strategies and predictive capabilities. One significant aspect of this is the development of virtual 3D models of patient anatomy. In neurosurgery, 3D images projected using AR technology allow surgeons to visualize complex procedures. Coelho et al. and Dubron et al. used AR as an educational tool to help novice surgeons become familiarized with the procedures, which Hey et al. believe minimizes risks [69-71]. Cannizzaro et al. agreed, adding that a unified educational plan for surgical training could be developed using AR [72]. However, AR technology remains expensive and is not easily accessible in certain regions. In the future, technology related to 3D imaging is expected to improve accuracy and cost-efficiency, making AR more accessible and widely available. The use of AR as an educational tool is also likely to continue evolving.

**Figure 3:**
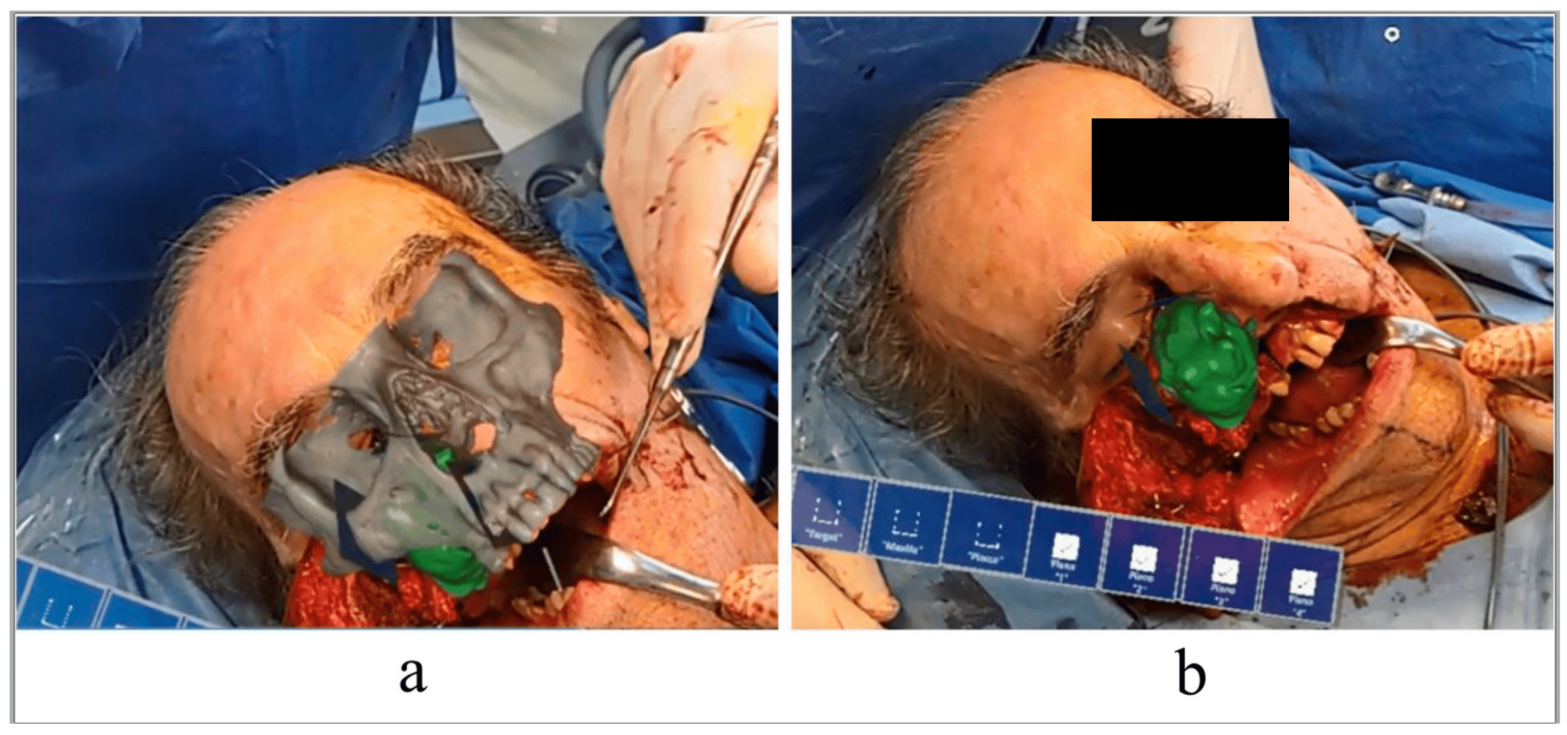
An example of augmented reality (AR) technology that projects a surgical plan onto a patient's face during surgery. Source: Ceccariglia et al. [23]. Used under the terms of the Creative Commons Attribution CC BY License.

For intraoperative applications, AR developments mainly focus on enhancing accuracy and precision while ensuring procedural safety. For instance, AR can help identify and visualize vulnerable patient structures to prevent errors from incorrect tool placement [73]. This is supported by the work of Louis et al. and Zhu et al., who developed SyncAR and a neuroendoscopic navigation system, respectively. These systems have assisted surgeons in locating delicate anatomy [74,75]. SyncAR demonstrated time reduction, assistance in clip positioning, and improved surgeon focus, achieving a 29% reduction in procedure time for aneurysms and being beneficial for clip positioning in 92.3% of cases. Meanwhile, the neuroendoscopic navigation system enhanced tool placement precision, with an average distance error of 1.28 mm, an average angle error of 1.34*, and a variance of 0.45*, proving high accuracy and feasibility. Ran et al. took a slightly different approach by developing a YOLOv7x model for surgical tool counting and detection, which achieved a higher accuracy when compared to traditional methods, with F1, AP, AP50, and AP75 reaching 4.6%, 3.1%, 3.6%, and 3.9% higher than traditional methods, respectively [76]. Martin-Gomez et al. similarly developed a surgical tool-tracking AR device that achieved an accuracy of approximately 0.1 mm on the lateral axis and 0.5 mm on the depth axis [77]. In spinal surgery, Molina et al. and Liu et al. used AR to assist in the placement of pedicle screws, achieving overall accuracies of 100% and 98%, respectively, as measured by the Gertzbein-Robbins scale [78,79]. In oncology procedures, AR allows surgeons to accurately detect and locate lesions, guide resections, and make precise incisions, thereby reducing intraoperative and postoperative complications, as demonstrated by Ceccariglia et al. [23,80]. In the future, developments relating to surgical navigation will likely continue as it provides the most potential to ensure surgical safety (Figure 3 and Table 3).

**Table 3:** Summary of AR applications in surgery. AR: augmented reality; MTCNN: multi-task cascaded convolutional neural network; vSLAM: visual simultaneous localization and mapping; API: application programming interface; CNN: convolutional neural network; ML: machine learning; MR: magnetic resonance; mAP: mean average precision.

Body part	Dataset	Method	Main results	Ref.
Brain	AR	D2P, model target function	Shows that a marker-less AR guidance system using Microsoft HoloLens 2 can be used to guide maxillary osteotomies.	[23]
ML surgical development	National Institute of India	MTCNN	Average media retrieval time (1.1471 μs) and client time (1.1207 μs) are much lower than the average API process time (288.934 ms).	[67]
	M2CAI 2016 challenge dataset	vSLAM, Mask R-CNN	10x faster than methods using regional CNN, with mean Jaccard score = 97.5, and mAP = 96.8 for tool detection.	[68]
	OSI26	YOLOv7x, CNN	Higher accuracy and robustness in surgical instrument detection tasks than traditional methods F1, AP, AP50, and AP75 reached 4.6%, 3.1%, 3.6%, and 3.9% higher than the baseline, respectively.	[76]
	Johns Hopkins University	Unity 3D, Direct X	Tracking accuracy of approximately 0.1 mm on the lateral axis and 0.5 mm on the depth axis.	[77]
	Tianjin University	Vector closed-loop algorithm	The average error of distance is 1.28 mm, avg. error of angles is 1.34*, and variance is 0.45*. Overall high accuracy and feasibility.	[75]
	Surgeons from five hospitals	NordicBrainEx, StealthStation S8	This resulted in a 29% reduction in time for aneurysms, useful for clip positioning in 92.3% of cases, and all surgeons reported better focus.	[74]
Spine	Patients from Johns Hopkins Hospital	None	100% clinical accuracy as measured by the Gertzbein-Robbins scale. Technical precision analysis resulted in a mean of 2.07 mm and an angular deviation of 2.41 degrees.	[78]
	Patients from Thomas Jefferson University Hospital	None	Screw placement accuracy of 98% overall as measured by the Gertzman-Robbins scale.	[79]
Education	Senior surgeon from Santa Marcelina Hospital	None	AR is adequate for educational purposes and shows potential for future applications.	[69]

Virtual reality in surgery

In contrast to AR, VR does not allow users to see their surrounding environment, which has limited its intraoperative applications in surgery. Additionally, VR development has been slower compared to AR due to challenges such as high costs and power consumption, resulting in fewer medical applications [81,82]. Current VR developments in surgery primarily focus on simulations for surgical training and preoperative planning [83]. The use of VR for surgical training is evident across various medical specialties, such as neurosurgery, orthopedics, hepatology, and laparoscopic surgery. For example, in maxillofacial surgery, Kim et al. used a VR simulation for preoperative planning, which allowed them to determine the osteotomy line while considering anatomical structures, safety margins, and accessibility of the instruments used during surgery [84]. Bartella et al. developed a VR application for preoperative planning, which enhanced procedural performance and improved spatial perception [85]. VR simulations have also been designed to facilitate collaborative planning as well, as demonstrated by Chheang et al., who created a VR environment where surgeons can collaborate to plan the removal of a tumor. All surgeons interviewed expressed high satisfaction and noted improved spatial perception [86]. Future developments in VR applications for preoperative planning are likely to include an improvement in graphics, more intuitive interfaces, and greater flexibility for collaboration.

Recent developments in VR have also focused extensively on generating simulations for educational purposes. Jacobsen et al. demonstrated that VR simulations for cataract surgery training showed a significant positive correlation between simulation performance and real-life procedural performance during the procedure (P = 0.003, R2 = 0.42) [87]. Overall, VR simulations for educational purposes have proven effective, showing a positive correlation between simulation practice and actual surgical outcomes. Currently, these VR simulations depend on surgical instructors to provide automatic feedback and primarily offer a space for novice surgeons to practice. However, Rogers et al. suggested that this limitation could be easily solved by incorporating ML into simulations [88]. Consequently, VR simulations are expected to be enhanced to include automated feedback after each simulation using ML, which will likely lead to greater integration into the surgical training curriculum (Table 4).

**Table 4:** Summary of VR applications in surgery. VR: virtual reality.

Body part	Dataset	Method	Main results	Ref.
Brain	Chosun University	Unity 3D	Was able to determine the osteotomy line and take into consideration anatomical structures, safety margins, and accessibility of the instruments used in the actual surgery.	[84]
	Aachen University	Open GL	Improved overall performance of the surgery, especially in terms of determining the relations to vital structures.	[85]
Heart/Lungs	Surgeons from the Otto-von-Guericke University	VR	Surgeons who used the application reported high satisfaction and a better spatial understanding.	[86]
Education	Surgeons from the University of Copenhagen	None	Conducted a study to establish the correlation between simulation performance and real-life procedure performance. Positive, significant correlation (P = 0.003, R2 = 0.42).	[87]

Mixed reality in surgery

Within preoperative and interventional planning, MR has primarily been evaluated for its effectiveness of visualization, depth perception, and image analysis to assist with complex surgical procedures [89,90]. In 2021, Dho et al. investigated the clinical application of patient-specific 3D-printed brain tumor models for neurosurgery and validated their clinical utility through electronic studies, reporting a p-value of less than 0.05 and noting that 18.8% of cases had their surgical goals modified according to the model [91]. Two studies on cardiovascular surgery examined the improvement of image visualization for diagnosis, showing significant time reductions in surgical preparation compared to two-dimensional imaging [92,93]. In addition to time reduction, Gehrsitz et al. also reported an improvement in depth perception (4.7 ± 0.7 vs. 3.7 ± 1.2), while Ye et al. found a 5.8% increase in diagnostic accuracy [92,93]. Moreover, the mental workload is significantly reduced for surgeons without the need for the psychological construction of images [92]. Similarly, in a study on the feasibility of using head-mounted displays (HMDs) for neuronavigation, Qi et al. found an overall median deviation of 4.1 mm, with 81.1% of lesions being highly consistent with standard neuronavigation (SN) (deviation < 5.0 mm), demonstrating both technical feasibility and accuracy [94]. For tumor removal, research indicates that using 3D holograms on HMDs results in statistically better tumor localization and surgical planning than computer images [95,96]. Throughout the past five years, the development of preoperative planning using MR significantly improved image processing, reduced workload, and enhanced detection and prediction. However, further development is needed to improve the accuracy and clinical efficacy of HMD systems for navigation purposes.

In intraoperative surgery, multiple studies have demonstrated that the application of MR is practical, reducing workload when dealing with complex anatomical structures and increasing accuracy and precision [97,98]. Wierzbicki et al. reported a significant reduction in intraoperative surgical time by one-third and a decrease in hospitalization duration by approximately four days when using 3D mixed reality as a support tool [95]. In a study on orthopedic surgery, results also showed improved effectiveness, spatial awareness, and better communication and understanding in surgeons for surgical work [99]. Similarly, in a study done by Zhu et al., the application of MR in hepatectomy was found to create shorter operation times (202.86 ± 46.02 minutes vs. 229.52 ± 57.13 minutes, P = 0.003) and reduced portal vein obstruction time (17.71 ± 4.16 minutes vs. 21.58 ± 5.24 minutes, P = 0.019) [6]. MR has the advantage of combining elements of VR and AR by allowing for easier interaction between the surgeon and the virtual surroundings and can be adapted for use in telemonitoring and control [100,101]. The development of MR for intraoperative use parallels advancements in preoperative surgical planning, with significant improvements in the application of 3D visualization to reduce workload and increase accuracy. Future directions for MR applications will likely focus on reducing learning curves and expanding application capabilities in intraoperative use to further minimize risk (Table 5).

**Table 5:** Summary of MR applications in surgery. MR: mixed reality; MRN: mixed-reality neuronavigation; SN: standard neuronavigation; CR: cinematic rendering; HMDs: head-mounted displays.

Body part	Dataset	Method	Main results	Reference
Brain	Patients from PLA General Hospital	Registration MRN system	Require 36.3 ± 6.3 minutes of additional time. The overall median deviation was 4.1 mm and 81.1% of the lesions were found to be highly consistent with SN (deviation < 5.0 mm).	[94]
	Chungbuk National University	None	Reported the usefulness of around 4.5/5 and 18.8% of cases modified their surgical goal.	[91]
Heart/Lungs	University Hospital Erlangen	Cinematic rendering	The average time required to create a CR hologram was 9.0 ± 2.1 minutes. Superiority over 2D and 3D printing and imaging. Less than 10+ minutes of surgical time.	[92]
	Chinese People's Liberation Army General Hospital	None	Diagnostic accuracy for the malformation was 5.8% higher. Surgical planning time was shorter for the variable group (51.65 ± 11.11 min).	[93]
	Ehime University Graduate School of Medicine	None	Found 3D hologram on HMDs than using the computer images (P < 0.01).	[97]
	Neo Hospital	CarnaLife Holo	Reduction of surgical time by ⅓ and shorter hospitalization period.	[96]
	Patient from First Teaching Hospital of Tianjin	None	A shorter operation time (202.86 ± 46.02 min vs. 229.52 ± 57.13 min, P = 0.003) and a shorter obstructive time of the portal vein (17.71 ± 4.16 min vs. 21.58 ± 5.24 min, P = 0.019).	[98]
	Johns Hopkins University	None	Improved accuracy by over 35% and ranked high based on usability	[100]
Education	Surgeons from the University of California San Diego	None	Surgeons reported that ARTEMIS successfully supported remote surgical collaboration.	[101]
Orthopedics	Huazhong University of Science and Technology	None	Using the NASA Task Load Index and Likert scale questionnaire, results showed improved understanding, communication, temporal, and spatial awareness.	[101]

Challenges and proposed solutions

Despite the rapid development of AI and ML technology for research purposes, XR still faces multiple challenges and limitations in its applications. One major issue is the need for larger and more suitable datasets for extended reality research in surgery, which is currently hindered by data privacy concerns and restrictions [17]. Comprehensive datasets encompassing aspects such as 3D visualization, real-time video, surgical tools, classification, and segmentation in ML are scarce, limiting proper testing. For this reason, to support future research, this article incorporates multiple tables to provide valuable insights and data summaries. With further development and broader adoption of XR, more comprehensive and suitable datasets are expected to emerge, better supporting ML training in XR while upholding enhanced data privacy standards. However, a persistent challenge in the ML field, including XR, is the “black box” problem, which continues to occur and shows the technology’s current limitations in achieving clinical readiness [102-104]. The lack of transparency and interpretability in AI applications causes mistrust among the devices, surgeons, and patients, posing a barrier to acceptance and clinical integration. Asking surgeons to accept a device in replacement of personal knowledge may be a struggle, especially with no prior knowledge of the functionality of the device. It could also trigger ethical concerns [17,105]. The “black box” problem can be mitigated through clinical trials and by providing thorough information to surgeons and hospitals. Additional measures could be taken as well, such as training surgeons and medical students to accurately identify situations where AI is beneficial and where it is not. Medical institutions and professional societies should promote increased collaboration between researchers and surgeons to achieve a deeper understanding that is closely tied to the patient's needs [103].

Through analyzing current trends, AR and MR hold a larger advantage for intraoperative surgical functions, whereas VR remains primarily limited to preoperative training and surgical preparation. VR also faces additional limitations, leading to a decrease in its research focus since the initial applications of XR, which suggests that this trend may likely continue as development progresses. Undeniably, as access to technology improves and solutions to the black box problem are developed, the applications of XR in medical settings and surgery will increase to guarantee further accuracy and precision while reducing risk and workload. The technicality of XR devices remains important not only for successful clinical trials, but also to reduce risk, machine errors, and machine bias. Further development is still necessary to reduce the black box problem and increase application accuracy and ability to further minimize risks.

## Conclusions

Over the past five years, AR, MR, and VR have been proven to be effective tools for assisting in the preoperative and intraoperative stages of surgery. Developmental trends in XR mainly focus on reducing workload and minimizing surgical errors through navigation, education, and visualization. It is evident that the use of XR and AI in medical applications will continue to increase with greater access to technology. In AR, developmental trends have largely pointed toward two main directions: navigational assistance during intraoperative guidance and the use of virtual 3D images as educational tools during preoperative planning. Over the last five years, AR has reduced surgical time by approximately 30% and has achieved an overall accuracy in intraoperative guidance and precision by approximately 95%.

MR developments have focused on improving diagnosis accuracy during the preoperative stage, reducing workload, and increasing accuracy in the intraoperative stage. Over the past five years, diagnosis accuracy using MR has improved by approximately 6%, and operation times have been reduced by five to 10 minutes. Lastly, VR technological advancements in surgery have primarily been directed toward the utilization of VR as an educational tool, with simulations of surgical scenes and procedures that allow novice surgeons to familiarize themselves with complex procedures. In the past five years, VR applications in surgical education have reduced operation time by around 40 minutes. While VR usage in surgery has been less prevalent, as many of VR’s functions can be easily substituted by AR, VR simulations for surgical education have gained popularity due to their potential to reduce surgical errors. AR and MR applications have been increasing in both popularity and number of applications due to their similar benefits, and they are expected to continue to improve their capabilities to accurately diagnose, predict, and guide. Specifically, developments in surgical navigation that support the removal or insertion of material are likely to advance further, as these complex tasks require the highest level of precision. VR applications for educational simulations will likely develop to be increasingly realistic and detailed. However, VR’s current limitations have resulted in fewer applications in the medical field, and until these limitations are resolved, future VR development in medical contexts is expected to remain limited.
